# Decreased efficacy of antimicrobial agents in a polymicrobial environment

**DOI:** 10.1038/s41396-022-01218-7

**Published:** 2022-03-19

**Authors:** Thomas James O’Brien, Wendy Figueroa, Martin Welch

**Affiliations:** Department of Biochemistry, University of Cambridge, Cambridge, United Kingdom

**Keywords:** Clinical microbiology, Antibiotics

## Abstract

The airways of people with cystic fibrosis (CF) often harbour diverse polymicrobial communities. These airway infections can be impossible to resolve through antibiotic intervention, even though isolates of the individual species present are susceptible to the treatment when tested in vitro. In this work, we investigate how polymicrobial cultures comprised of key CF-associated pathogens respond to challenge with species-specific antimicrobial agents; colistin (targets *Pseudomonas aeruginosa*), fusidic acid (targets *Staphylococcus aureus*), and fluconazole (targets *Candida albicans*). We found that growth in a polymicrobial environment protects the target microorganism (sometimes by several orders of magnitude) from the effect(s) of the antimicrobial agent. This decreased antimicrobial efficacy was found to have both non-heritable (physiological) and heritable (genetic) components. Whole-genome sequencing of the colistin-resistant *P. aeruginosa* isolates revealed single nucleotide polymorphisms and indels in genes encoding lipopolysaccharide (LPS) biosynthesis and/or pilus biogenesis, indicating that a previously undescribed colistin resistance mechanism was in operation. This was subsequently confirmed through further genetic analyses. Our findings indicate that the polymicrobial nature of the CF airways is likely to have a significant impact on the clinical response to antimicrobial therapy.

## Introduction

A common approach to manage infections is through antimicrobial intervention. Here, the susceptibility of the infecting microorganism to antimicrobial treatment is assessed by determining the minimum inhibitory concentration (MIC) of clinical isolates during planktonic growth in vitro using Clinical and Laboratory Standards Institute (CLSI) guidelines. With this information in hand, the antibiotics most likely to be effective on the particular isolate are chosen. The problem comes when the infection is not cleared by treatment(s) that are effective in vitro. In the past few decades there have been an increasing number of reports of infections that are apparently impossible to treat with traditional antimicrobial therapy [1]. Although many such cases are linked to multi-drug resistant (MDR) strains of bacteria, some are associated with microorganisms that show susceptibility to antibiotics in vitro and nevertheless are not killed when treated in vivo.

Microbial antibiotic resistance is relatively common in isolates obtained from people with cystic fibrosis (CF), a disease caused by defective activity or targeting of the cystic fibrosis transmembrane conductance regulator (CFTR). The resulting defect(s) in CFTR function leads to the overproduction of nutrient-rich mucilaginous secretions in the airways, and this predisposes people with CF to life-long infections. These infections are often polymicrobial and include bacteria such as *Pseudomonas aeruginosa* (PA) and *Staphylococcus aureus* (SA) [2], as well as fungal species [3–6]. However, and despite the aggressive antimicrobial treatment(s) used to manage chronic CF airway infections, the colonising microorganisms often survive.

There is increasing evidence that interactions between bacterial pathogens and other co-habiting microbes at the site of infection can modulate the gene expression profile and virulence of the pathogen [7–12]. Indeed, we recently showed that species comprising as little as 0.05% (by cell numbers) can significantly alter the production of extracellular molecules by a more abundant species in the mixture [13]. It therefore seems very likely that interspecies interactions may also alter the response of pathogens, such as PA, to antimicrobial agents [14–17]. This notwithstanding, traditional antimicrobial susceptibility testing does not take into account the presence of co-habiting species, nor does it consider how such microorganisms might affect the acquisition of resistance to antibiotics.

Even though some microbial species are known to co-habit in the human airways, there is no guarantee that these species can be stably co-cultivated in ex situ models [18]. To redress this issue, we recently introduced a continuous flow model that allows PA, SA, and *Candida albicans* (CA) to be maintained in artificial sputum medium in a remarkably stable steady-state [13]. In the current study we examine the effects of three clinically relevant, species-specific antimicrobials (colistin, fusidic acid, and fluconazole) on steady-state polymicrobial populations containing PA, SA, and CA. We find that all three species-specific antimicrobials demonstrate lower activity against their target microorganism in the polymicrobial cultures, compared with single-species cultures. We also identify a novel colistin resistance mechanism in PA that arises at higher frequency in the presence of other species. Taken together, our findings highlight the need to consider inter-species interactions when deploying antimicrobial agents in polymicrobial infection scenarios, and further reinforce the notion that rare/low abundance species may have a profound effect on the physiology and behaviour of the other co-habitants in the niche.

## Materials and methods

### Microbial strains and growth media

The bacterial/fungal strains used in this study were: *P. aeruginosa* PAO1 (PA; strain MPAO1 from Colin Manoil), *S. aureus* ATCC 25923 (SA), and *C. albicans* SC314 (CA). Artificial sputum medium (ASM) was freshly prepared for every experiment as described in [19].

### Culture conditions

The continuous-flow culture vessel was assembled, inoculated and incubated as described in [13]. We also refer the reader to [19] for an illustrated, step-by-step guide on the assembly and use of the continuous-flow model. In brief, the continuous-flow culture vessel comprises a 100 mL Duran bottle fitted with a 4-port HPLC GL80 screwcap lid. The liquid contents of the culture vessel are kept homogenous with gentle stirring (100 rpm). A multi-channel peristaltic pump is used to deliver fresh medium (ASM) into the culture vessel from a reservoir, and to remove spent medium at the same continuous rate of flow (*Q*). Unless otherwise indicated, each species was introduced into the culture vessel to achieve a starting OD_600_ of 0.05. The vessel was incubated for 3 h prior to staring the flow of medium. For experiments not containing CA, *Q* was set at 170 µL min^−1^. For experiments including CA, *Q* was decreased to 145 µL min^−1^. Samples (1 mL volume) were withdrawn, and antimicrobials were added to the culture using a syringe fitted with a sterile needle inserted through the rubber septa in the HPLC ports. Batch cultures were set up in the same way, except that *Q* = 0 µL min^−1^. For all experiments, the culture temperature was maintained at 37 °C.

### Quantitative real-time PCR (RT-PCR)

Cells were harvested from continuous-flow or batch cultures by removing 2 mL of the culture and pelleting *via* centrifugation (13,000 × *g*, 1 min, 4 °C). RNA was extracted using an RNeasy Plus Mini Kit (Qiagen) following the manufacturers’ bacterial extraction protocol. cDNA synthesis was performed in a single-step reaction using the High-Capacity cDNA Reverse Transcription Kit (ThermoFisher) following the manufacturers’ instructions. Four PA transcripts that are known to be strongly up-regulated during exponential growth and four transcripts that are known to be up-regulated during the stationary phase were selected for quantification. These transcripts were chosen based on a previously published transcriptomic dataset [20]. Primers and reaction conditions for real-time PCR (RT-PCR) were designed using the guidelines described in [21] (Supplementary Tables S1 and S2). Cycle threshold (C_t_) values of cDNA samples obtained by RT-PCR were analysed using the comparative ΔΔC_t_ method [22]. For each target gene, C_t_ values were normalised according to the C_t_ value of the constitutively expressed 16S rRNA housekeeping gene [23] on the same reaction plate.

### Determination of antimicrobial minimum inhibitory concentrations (MICs)

The minimum inhibitory concentration (MIC) of colistin, fluconazole, and fusidic acid was determined in ASM using the broth microdilution method described by the Clinical and Laboratory Standards Institute (CLSI, 24) [24]. Briefly, serial 2-fold dilutions of each antimicrobial agent were made in fresh, pre-warmed ASM and dispensed into a 96-well microtiter plate (Nunc). Overnight cultures of each microbial strain were washed three times in sterile PBS and used to inoculate the wells to an initial OD_600_ of 0.05. The final volume of liquid in each well was 150 µL. The plates were sealed with a gas permeable Breathe-Easy membrane (Sigma) and incubated for 16 h at 37 °C with 100 rpm shaking. The MIC was taken as the lowest antimicrobial concentration able to inhibit visible microbial growth.

### CFU mL^−1^ enumeration

Colony-forming units (CFU) per mL of culture were determined using the single plate-serial dilution spotting (SP-SDS) method [25] on selective agar media, as described in [13].

### Exposure of steady-state communities to antimicrobial compounds

Mono-species or triple-species cultures of *P. aeruginosa* PAO1, *S. aureus* 25923, and *C. albicans* SC5314 were grown until they reached a steady-state (24 h post-inoculation) under continuous-flow conditions. These cultures were then treated with the appropriate antimicrobial compound at a final concentration of 2× or 5× MIC. The antimicrobial agent was introduced *via* a sterile needle inserted through the rubber septum in the HPLC port. Cultures were then incubated for 1 h with no flow (*Q* = 0 µL min^−1^). An appropriate flow rate, *Q* = 170 µL min^−1^ for PA and SA mono-species cultures and *Q* = 145 µL min^−1^ for CA mono-species and triple-species cultures, was then applied to the culture vessel. Unless otherwise indicated, the setup was incubated for a further 48 h. Stock concentrations of colistin (10 mg mL^−1^ in water, Sigma), fluconazole (10 mg mL^−1^ in ethanol, Sigma), and fusidic acid (10 mg mL^−1^ in ethanol, Sigma) were made fresh prior to each experiment. Control cultures were treated with an equal volume of water or ethanol (used to dissolve the antimicrobials) but showed no change in the cell titres of any species following the addition of the solvent (Supplementary Fig. S1).

### Whole-genome sequencing (WGS)

Eight PA isolates able to grow in the presence of 1 mg mL^−1^ colistin were selected for whole-genome sequencing (WGS). These eight isolates were randomly selected from the set of colistin-resistant isolates obtained after challenge of the cultures with 2× MIC_colistin_. As a control, we also carried out WGS on the progenitor strain, MPAO1, used to inoculate the continuous-flow culture vessel. Two resistant isolates were selected from: the mono-species culture after 1 h exposure to 8 µg mL^−1^ colistin (isolates A and B); the mono-species culture after 8 h exposure to 8 µg mL^−1^ colistin (isolates E and F); the polymicrobial co-culture after 1 h exposure to 8 µg mL^−1^ colistin (isolates C and D), and the polymicrobial co-culture after 8 h exposure to 8 µg mL^−1^ colistin (isolates G and H). WGS of the isolates was performed by MicrobesNG (Birmingham, UK) using the Nextera XT library prep protocol v.05 (Illumina) on an MiSeq platform (Illumina) using 2 × 250 bp paired-end reads. The reads were adaptor-trimmed using Trimmomatic v0.30 with a sliding window quality cut-off of Q15. Taxonomic classification of sequences and assessment of sequence contamination was performed using Kraken [26]. The *de novo* assembly of contigs was performed using SPAdes v3.14.0 with the default parameter settings and automated annotation of the resulting contigs was performed using Prokka v1.12. Variants were called using Snippy v2.5/Freebayes v0.9.21-7 (https://github.com/tseemann/snippy) with a minimum base quality of 20, read coverage of 10× and a 90% read concordance at a locus for a variant to be reported. Variants in the different isolates were compared against the progenitor strain (MPAO1) to determine mutations likely selected as a consequence of treatment with 8 µg mL^−1^ colistin. Called variants were visually inspected by mapping the reads on the reference genome (accession number NC_002516) using the software Artemis (http://sanger-pathogens.github.io/Artemis/Artemis/).

### Cloning and complementation

The wild-type *wzy* ORF was PCR-amplified from wild-type MPAO1 genomic DNA template (Supplementary Tables S3 and S4). The PCR product was purified using a GeneJET PCR Purification Kit (Thermo Scientific) and cloned into plasmid pUCP20 plasmid at the restriction sites SacI and SphI. Electrocompetent *E. coli* DH5α cells were prepared and electroporated following the protocol reported in [27]. Transformants were confirmed by PCR and the plasmid was extracted using a GeneJET Plasmid Miniprep Kit (Thermo Scientific). Electrocompetent cells of *Pseudomonas aeruginosa* (MPAO1, isolate D, and isolate F) were prepared following the protocol described in [28]. Complementation of the colistin-resistant phenotype was confirmed by measuring the minimal inhibitory concentration (MIC) of colistin against MPAO1, isolates D and F alone, and against these strains containing the empty vector or containing pUCP20-*wzy*. After incubation overnight, 10 µL of 0.02% resazurin was added to each well of the MIC plates to detect cell viability and determine the minimal inhibitory concentration of colistin.

### Bioinformatics analysis

The International *Pseudomonas* Consortium Database (IPCD) (comprising 854 *Pseudomonas aeruginosa* whole-genome sequences) was downloaded from GenBank (accession number PRJNA325248). Multi-locus sequence typing (MLST) and the sequence type (ST) of the strains in the database were determined using the software mlst (https://github.com/tseemann/mlst). Variants for each genome in the IPCD were called as previously described in the section on whole-genome sequencing (WGS). The called variants in the genes *wbpA*, *wbpE*, and *wzy* were then extracted from the variant calling files and deposited together in a new table. The metadata associated with the genomes was extracted using Entrez Direct in Unix command line [29]. Analysis and visualisation of mutations and associated metadata were performed in R 3.4.0.

### Statistical analysis

Unless otherwise stated, all data represent the mean ± SD of three independent biological experiments. Statistical differences in CFU mL^−1^ counts between single-species cultures were analysed by one-way ANOVA and differences in CFU mL^−1^ counts between microbial co-cultures were analysed by two-way ANOVA using GraphPad Prism version 8.2.0. Statistical analysis for the real-time PCR experiments was performed in R 3.4.0. To determine whether the data was normally distributed, Shapiro-Wilk tests were performed taking as input the expression values for each gene in each time point in the two different conditions (continuous-flow and batch culture). As the tests showed that data were normally distributed, Bartlett’s tests were used to assess whether the samples had the same variance. As the latter showed heterogenous variances, two-sided Welch’s T-test was used to calculate *p* values of gene expression in continuous-flow vs batch culture conditions. All *p* values lower than 0.05 were considered statistically significant.

## Results

### Physiological state of PA in the continuous flow setup

**Fig. 1:**
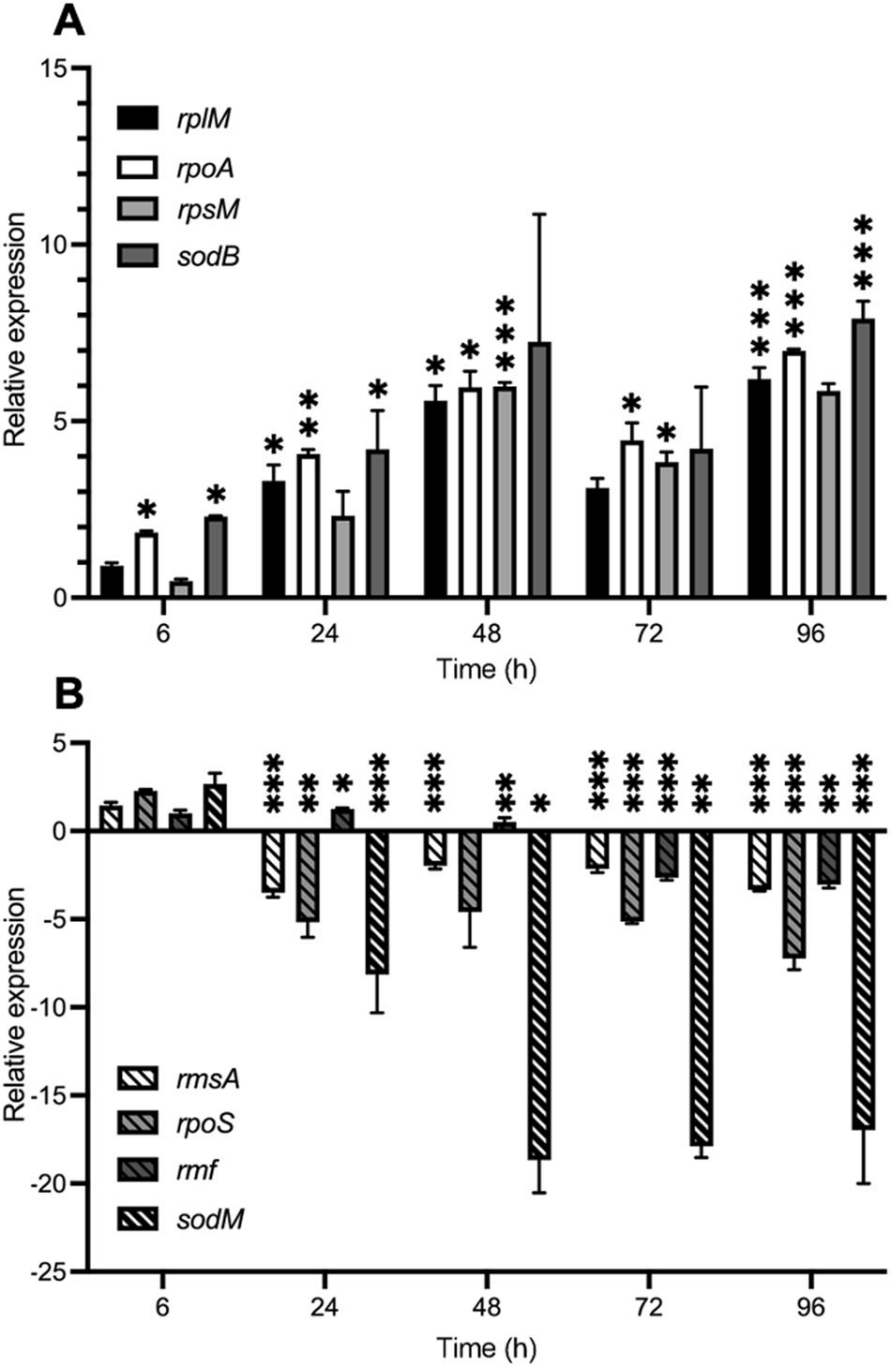
Relative expression of genes associated with exponential and stationary phase growth in PA. The figure shows the fold change expression of the indicated genes in *P. aeruginosa* PAO1 mono-species cultures grown in continuous-flow conditions (*Q* = 170 µL min^−1^) compared with stirred batch conditions (*Q* = 0 µL min^−1^). **A** Genes associated with exponential growth; **B** genes associated with the stationary phase. Cycle threshold (C_t_) values obtained by quantitative real-time PCR were normalised against expression of the 16S rDNA housekeeping gene and analysed using the comparative ΔΔC_t_ method. Positive values indicate greater expression in the continuous-flow culture; negative values indicate greater expression in the batch culture. Data are the mean ± standard deviation of three independent experiments. Asterisks represent significant differences in relative gene expression between cultures maintained under the different growth conditions (*p < 0.05, **p < 0.005, ***p < 0.001).

Most antimicrobial agents work best on actively growing cells. There is evidence to suggest that in the CF airways, the *P. aeruginosa* (PA) population is maintained in an actively-growing state [30]. We therefore wondered whether the PA in our continuous flow setup was similarly actively growing, or whether it was maintained in a condition more closely approximating to the stationary phase. Quantitative reverse-transcription PCR (RT-PCR) was used to monitor the relative expression of genes known to be associated with exponential phase- and stationary-phase physiology in PA. The expression level of each gene was measured in samples of axenically-grown PA taken from the continuous-flow setup and from a parallel batch culture. By 24 h, the continuous flow culture had reached a constant OD_600_ (indicative of steady-state growth). Similarly, the batch culture also had a constant OD_600_ (Supplementary Fig. S2). However, by 24 h, the expression of all genes associated with exponential phase growth (*rplM*, *rpoA*, *rpsM*, and *sodB*) was high in the continuous flow cultures (Fig. 1A), whereas the expression of genes associated with the stationary phase (*rmsA*, *rpoS*, *rmf*, and *sodM*) was high in the batch cultures (Fig. 1B). The relative expression of ribosomal modulation factor (encoded by *rmf*), which converts the 70S subunit into an inactive 100S dimer during the stationary phase, displayed a more gradual decrease over time, but nevertheless, has a negative relative expression value by the 72 h sampling point. Taken together, these results suggest that at carrying capacity, the microbial population in the continuous-flow culture vessel is maintained at a steady state in the exponential phase of growth.

### Antimicrobial minimum inhibitory concentrations in artificial sputum medium

Consistent with our previous study [13], axenic cultures of *Pseudomonas aeruginosa* PAO1 (PA), *Staphylococcus aureus* ATCC 25923 (SA), or *Candida albicans* SC5314 (CA), and polymicrobial co-cultures of all 3 species reached a steady-state with respect to viable cell counts by 24 h incubation, and there was no significant difference (*p* > 0.9) in CFU mL^−1^ counts of any species after this point (Supplementary Fig. S1A). We next determined the minimum inhibitory concentration (MIC) of three species-specific antimicrobial compounds against PA, SA, and CA grown artificial sputum medium (ASM) (Supplementary Table S5). These data confirmed that colistin (MIC_PA_ = 4 μg mL^−1^) has little activity against SA or CA, fluconazole (MIC_CA_ = 1 μg mL^−1^) has little activity against PA or SA, and that fusidic acid (MIC_SA_ = 15.6 ng mL^−1^) has little activity against PA or CA.

To examine the response of mono-species and polymicrobial cultures to antimicrobial agents, we challenged each these with 2× or 5× MIC of each antimicrobial agent. Following addition of the antimicrobial agent, stirring was maintained but the flow was turned off for 1 h to allow the compound time to act before dilution with fresh medium. After 1 h, the flow of fresh medium was restored at the original rate. As fresh ASM enters the culture vessel it gradually dilutes the antimicrobial agent (dilution rate, *D* = 8.7 × 10^−2^ and 0.102 h^−1^ for *Q* = 145 and 170 µL min^−1^, respectively), loosely mimicking the metabolism and excretion of antimicrobials when provided to a patient in situ.

### Treatment with colistin

**Fig. 2:**
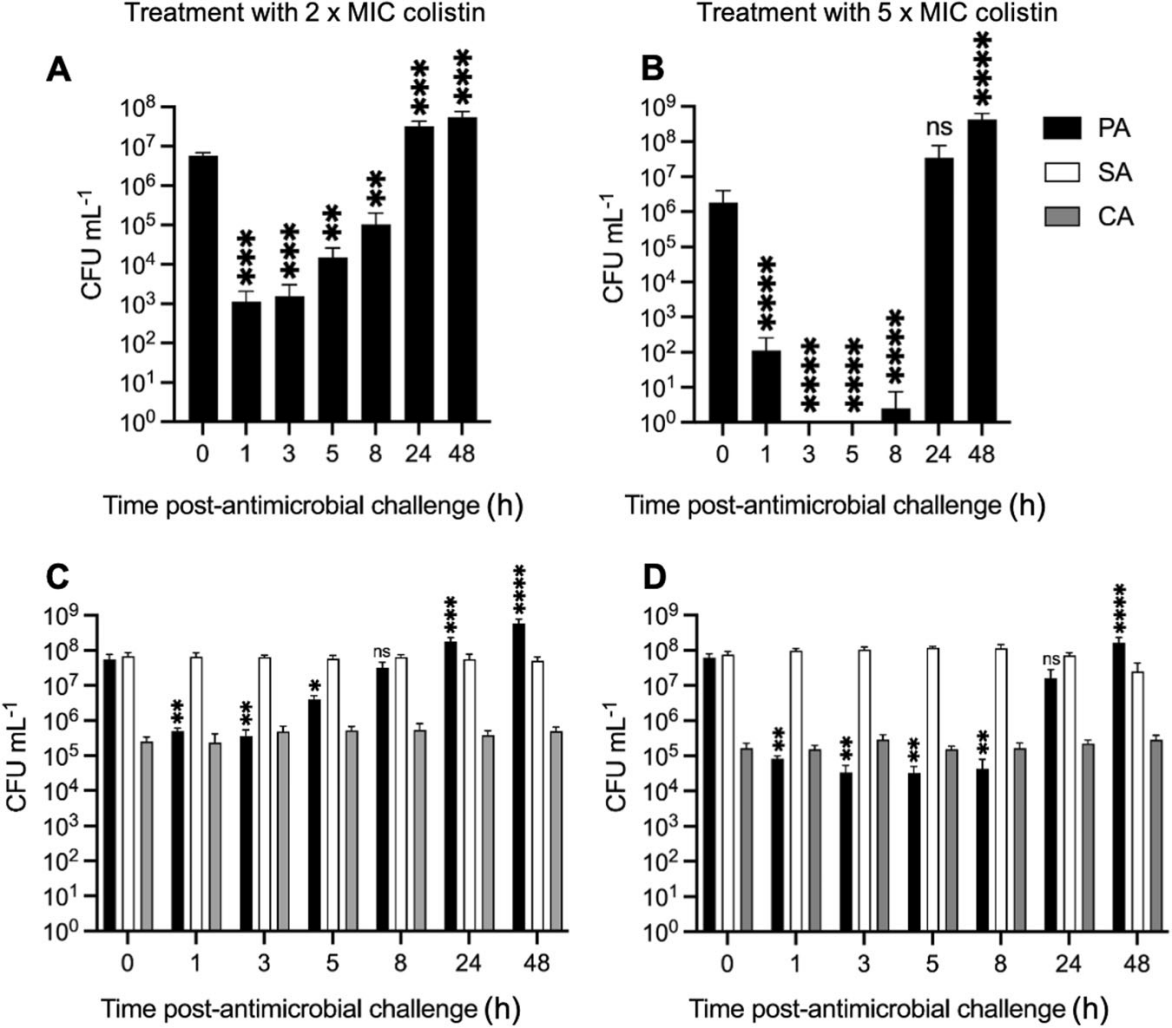
Perturbation of steady-state microbial cultures with 2× and 5× MIC colistin. Mono-species cultures of *P. aeruginosa* PAO1 (PA), and mixed-species cultures of *P. aeruginosa* PAO1 (black bars), *S. aureus* 25923 (white bars), and *C. albicans* SC5314 (grey bars) were grown to a steady-state in ASM under continuous-flow conditions (24 h incubation). Bars represent viable cell counts (CFU mL^−1^) in (**A**, **B**) mono-species PA, and (**C**, **D**) mixed-species PA/SA/CA populations following the addition of 8 µg mL^−1^ (2× MIC) or 20 µg mL^−1^ (5× MIC) colistin, as indicated, to the culture vessel at *T* = 0 h. Data represented as the mean ± standard deviation from three independent experiments. Asterisks represent significant differences in PA CFU mL^−1^ counts in comparison to counts at the *T* = 0 h time point (*p < 0.05, **p < 0.005, ***p < 0.001, ****p < 0.0001).

Pre-established *P. aeruginosa* (PA) mono-species and triple-species populations were challenged with 2× MIC (8 µg mL^−1^) or 5× MIC (20 µg mL^−1^) colistin (Fig. 2). Following 2× MIC colistin addition to the mono-species culture, PA titres declined 10^4^-fold over the first 3 h. After this, titres increased, and by 24–48 h post-colistin treatment had even reached significantly higher values than those immediately prior to challenge with the antimicrobial (Fig. 2A). A similar sharp decline in PA titres was observed in the mono-species culture treated with 5× MIC colistin, although here, by 3–5 h post-treatment, no detectable viable PA cells could be recovered from the culture vessels. However, the population rapidly recovered over the next 16 h, and by 48 h, PA titres were once again higher (*p* < 0.0001) than the steady-state viable cell counts prior to the addition of colistin (Fig. 2B).

Challenging polymicrobial cultures with 2× MIC colistin led to a 10^2^-fold decrease in PA titres over the first 3 h. This was a much smaller decrease in cell titres than the one observed in the mono-species culture. Following this, PA titres gradually increased, eventually becoming significantly higher than the starting (pre-treatment) titre (Fig. 2C). The impact of colistin was only marginally greater when the cultures were challenged with 5× MIC; following an initial 10^2^−10^3^-fold decrease in PA titres, the population had completely recovered by 24 h (Fig. 2D). We conclude that growth in a polymicrobial environment provides protection to PA against the bactericidal effects of colistin. We saw no significant change in *S. aureus* (SA) or *C. albicans* (CA) titres at any point of sampling following challenge with either 2× MIC or 5× MIC colistin. This suggests that when the niche normally occupied by PA in the polyculture becomes vacated (due to the action of colistin), this does not necessarily lead to an altered carrying capacity in the other species present.

### Treatment with fusidic acid

**Fig. 3:**
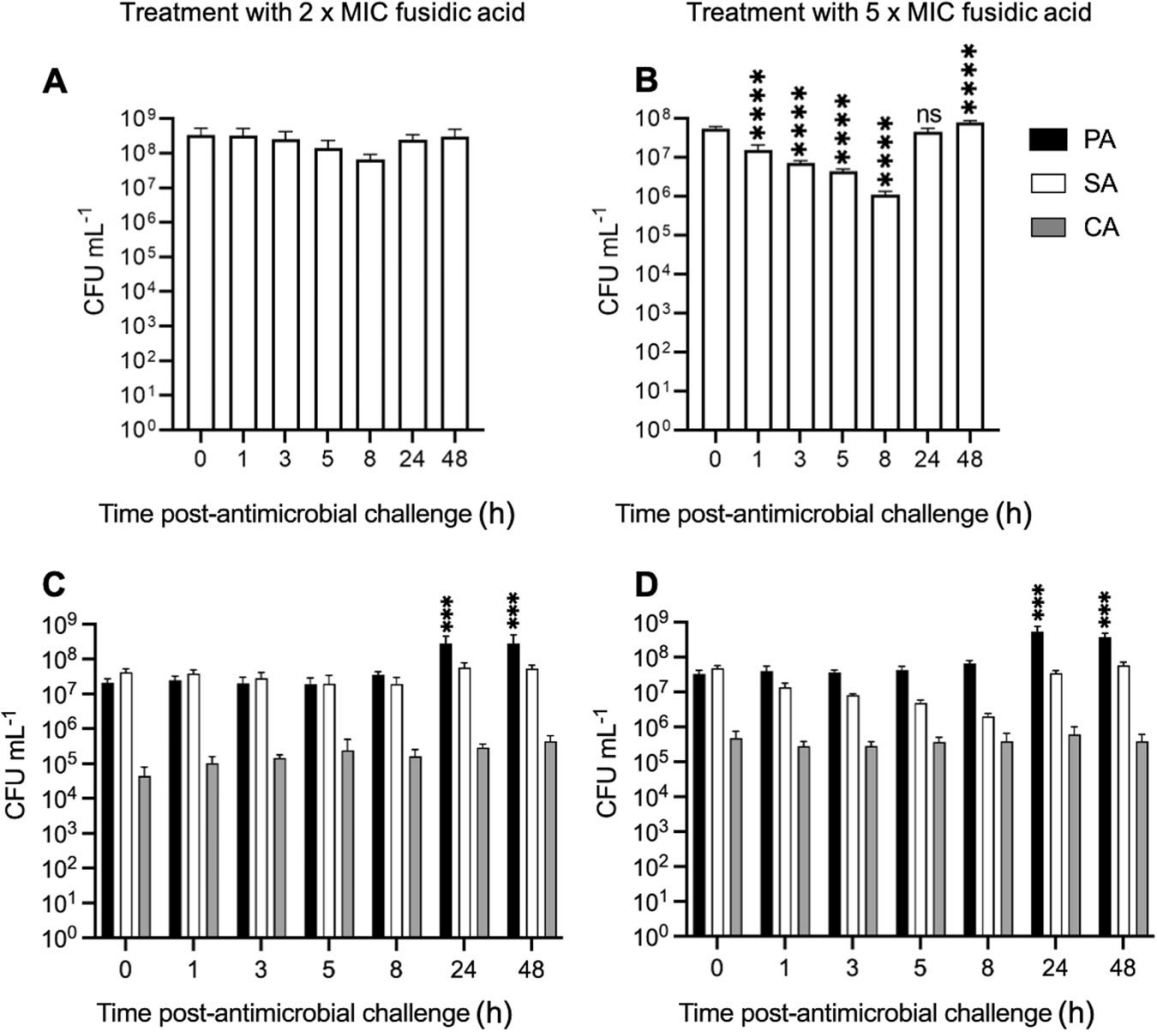
Perturbation of steady-state microbial cultures with 2× and 5× MIC fusidic acid. Mono-species cultures of *S. aureus* 25923 (SA), and mixed-species cultures of *P. aeruginosa* PAO1 (black bars), *S. aureus* 25923 (white bars), and *C. albicans* SC5314 (grey bars) were grown to a steady-state in ASM under continuous-flow conditions (24 h incubation). Bars represent viable cell counts (CFU mL^−1^) in (**A**, **B**) mono-species SA, and in (**C**, **D**) mixed-species populations following the addition of 31.2 ng mL^−1^ (2× MIC) or 78 ng mL^−1^ (5× MIC) fusidic acid (as indicated) at *T* = 0 h. Data represent the mean ± standard deviation from three independent experiments. Asterisks represent significant (***p < 0.001, ****p < 0.0001) differences in SA CFU mL^−1^ counts in comparison to counts at the *T* = 0 h time point. *p* < 0.05 is considered not significant (ns).

Mono-species and triple-species cultures containing *S. aureus* (SA) were challenged with 2× MIC (31.2 ng mL^−1^) and 5× MIC (78 ng mL^−1^) fusidic acid (Fig. 3). Interestingly, there was no change in SA titres in the mono-species culture following treatment with 2× MIC fusidic acid (Fig. 3A). This is presumably due to the progressive dilution of this bacteriostatic antibiotic in the setup. However, the effect of fusidic acid on the mono-culture was more pronounced when applied at 5× MIC, with a ca. 10^2^-fold decrease in SA titres apparent between *T* = 0 h and 8 h (Fig. 3C). SA titres then recovered, and by 48 h post-treatment had even surpassed the pre-treatment level by around 5-fold (*p* < 0.0001).

There was no statistically significant change (*p* > 0.8) in *S. aureus* (SA) or *C. albicans* (CA) titres across any time points for the polymicrobial culture treated with 2× MIC or 5× MIC fusidic acid (Fig. 3B, D). However, we did observe a significant (*p* < 0.001) ca. 10-fold increase in *P. aeruginosa* (PA) titres following challenge with 2× MIC_fusidic acid_ and 5× MIC_fusidic acid_ when comparing the *T* = 0 h and *T* = 24 h samples. This observation suggests that even minor perturbations in the composition or physiology of the non-PA microbial community – in this case, driven by the addition of fusidic acid – may be sufficient to trigger a change in PA titres.

### Treatment with fluconazole

**Fig. 4:**
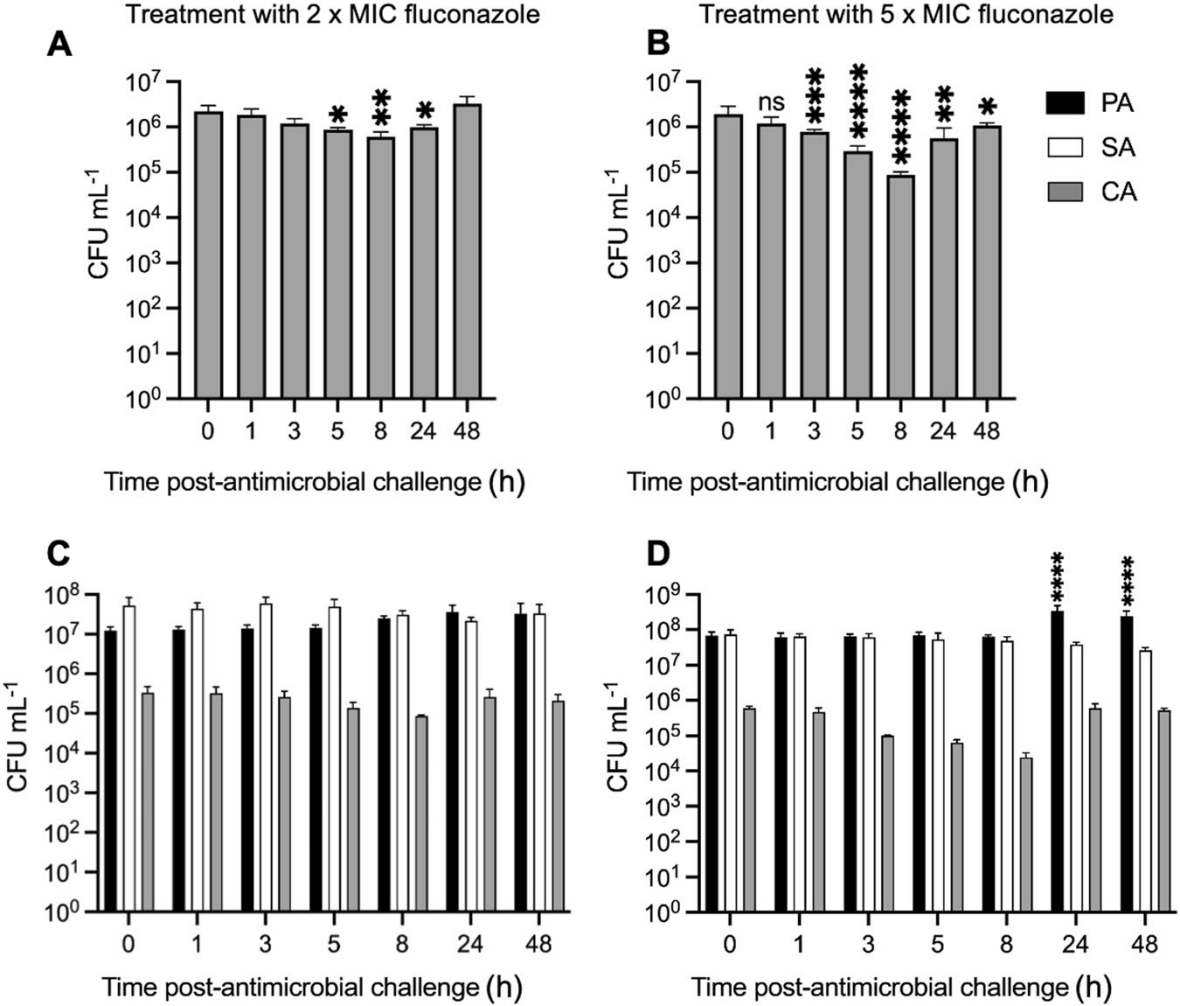
Perturbation of steady-state microbial cultures with 2× and 5× MIC fluconazole. Mono-species cultures of *C. albicans* SC5314 (CA), and mixed-species cultures of *P. aeruginosa* PAO1 (black bars), *S. aureus* 25923 (white bars), and *C. albicans* SC5314 (grey bars) were grown to a steady-state in ASM under continuous-flow conditions (24 h incubation). Bars represent viable cell counts (CFU mL^−1^) in (**A**, **B**) mono-species CA, and (**C**, **D**) mixed-species populations following the addition of (**A**, **C**) 2 µg mL^−1^ or (**B**, **D**) 5 µg mL^−1^ fluconazole to the culture vessel at *T* = 0 h. Data represented as the mean ± standard deviation from three independent experiments. Asterisks represent significant (*p < 0.05, **p < 0.01, ***p < 0.001, ****p < 0.0001) differences in CA CFU mL^−1^ counts in comparison to counts at the 0 h time point. *p* > 0.05 is considered not significant (ns).

Pre-established *C. albicans* (CA) mono-species and polymicrobial populations were challenged with 2× MIC (2 µg mL^−1^) and 5× MIC (5 µg mL^−1^) fluconazole (Fig. 4). There was a significant (*p* < 0.01) ca. 10-fold decrease in CA titres recovered from the axenic population treated with 2× MIC fluconazole following the first 8 h of fluconazole treatment, although titres recovered to pre-treatment levels by the 48 h time point (Fig. 4A). There was no significant change in CA titres in the polymicrobial population treated with 2× MIC fluconazole.

Treatment of the mono-species CA culture with 5× MIC fluconazole led to a more pronounced effect, with a 100-fold decrease in CA titres 8 h after addition of the compound (Fig. 4C). Titres did recover over the next 40 h, although they remained significantly lower (*p* < 0.05) than the pre-treatment levels. Challenge of the polymicrobial culture with 5× MIC fluconazole also resulted in a decrease in CA titres, although this decrease was not significant (*p* > 0.05). However, and similar to the situation seen following challenge of the polymicrobial cultures with fusidic acid, we observed a significant (*p* < 0.001) ca. 10-fold increase in *P. aeruginosa* (PA) titres following challenge with 5× MIC fluconazole when comparing the *T* = 0 h and *T* = 24 h samples. This observation reinforces the notion that minor perturbations in the composition or physiology of the non-PA microbial community can trigger a change in PA titres.

Taken together, our findings demonstrate that growth in a polymicrobial consortium confers a high level of protection in sensitive species against the action of microbicidal (colistin) and microbiostatic (fusidic acid, fluconazole) agents. We also conclude that antimicrobial compounds that target non-PA species may confer a selective advantage (in terms of cell titres) on the PA present.

### MIC of recovered isolates

We next sought to determine whether the protection against antimicrobial agents conferred by growth in the presence of co-cultured species was heritable. Thirty-two isolates per experimental condition were randomly selected from agar spreads generated from both the axenic and polymicrobial cultures challenged with each different antimicrobial agent (applied at 2× and 5× MIC). Following colony purification, the resistance profile of each isolate was measured and compared against the MIC of the progenitor strain (Supplementary Table S5) used to inoculate the cultures (Supplementary Tables 1 and 2).

**Table 1:** Growth of recovered *P. aeruginosa* isolates in ASM with colistin.

Time (h)	MIC (µg mL^−1^) of colistin against isolates collected from mono-species culture challenged with:							
	2× MIC colistin				5× MIC colistin			
1	25	45	>1000	>1000	20	25	45	45
8	20	45	>1000	>1000	20	25	20	20
24	20	20	20	20	20	20	20	20
48	20	20	20	20	20	20	20	20
	**MIC (µg mL**^**−1**^**) of colistin against isolates collected from polymicrobial co-culture challenged with:**							
	**2× MIC colistin**				**5× MIC colistin**			
1	45	45	>1000	>1000	20	20	25	45
8	45	>1000	>1000	>1000	25	25	45	45
24	25	45	>1000	>1000	20	20	25	25
48	20	20	20	20	20	20	20	20

Number of *P. aeruginosa* PAO1 isolates able to grow in ASM containing the indicated concentration of colistin (in µg mL^−1^). Four isolates were collected at each time point (1 h, 8 h, 24 h and 48 h) after challenge of each culture with 2× or 5× MIC colistin (i.e., 4 × 4 × 2 = 32 isolates in all). Isolates were randomly selected from agar spreads of appropriately diluted single-species (upper panel) or PA/SA/CA mixed-species (lower panel) steady-state continuous-flow cultures. The ability of each isolate to grow in different concentrations of colistin was then determined. The results were confirmed over three independent experiments using fresh batches of ASM. Shades of grey indicate resistance to colistin relative to WT; dark grey < light grey < white.

**Table 2:** Growth of recovered *C. albicans* isolates in ASM with fluconazole.

Time (h)	MIC (µg mL^−1^) of fluconazole against isolates collected from mono-species culture challenged with:							
	2× MIC fluconazole				5× MIC fluconazole			
3	2.5	2.5	2.5	10	2.5	2.5	2.5	2.5
8	5	10	10	10	5	5	10	10
24	5	5	10	10	2.5	5	5	10
48	2.5	2.5	2.5	2.5	1	2.5	2.5	2.5
	**MIC (µg mL**^**−1**^**) of fluconazole against isolates collected from polymicrobial co-culture challenged with:**							
	**2× MIC fluconazole**				**5× MIC fluconazole**			
3	5	10	10	10	5	5	5	10
8	5	10	10	10	5	5	10	10
24	5	5	10	10	2.5	5	5	10
48	2.5	5	10	10	1	1	1	2.5

Number of *C. albicans* SC3541 isolates able to grow in ASM containing the indicated concentration of fluconazole (in µg mL^−1^). Four isolates were collected at each time point (3 h, 8 h, 24 h and 48 h) after challenge of each culture with 2× or 5× MIC fluconazole (i.e., 4 × 4 × 2 = 32 isolates in all). Isolates were randomly selected from agar spreads of appropriately diluted single-species (upper panel) and PA/SA/CA mixed-species (lower panel) steady-state continuous-flow cultures. The ability of each isolate to grow in different concentrations of fluconazole was then determined. The results were confirmed over three independent experiments using fresh batches of ASM. Shades of grey indicate resistance to colistin relative to WT; dark grey < light grey < white.

There was no difference between the MIC of the *S. aureus* (SA) progenitor strain and any SA isolate collected from either the mono-species or polymicrobial cultures treated with any concentration of fusidic acid (*data not shown*). This suggests that the diminished efficacy of fusidic acid against SA in polymicrobial cultures arises from a protective, not an adaptive (i.e., heritable), mechanism of resistance. However, this was not the case with the colistin-treated *P. aeruginosa* (PA). Here, all of the tested PA isolates from the polymicrobial and mono-species cultures challenged with either 2 × MIC_colistin_ or 5 × MIC_colistin_ could grow on at least 5 × MIC_colistin_ (Table 1). Indeed, and remarkably, 7/32 (22%) of the PA isolates from the polymicrobial cultures and 4/32 (13%) of the PA isolates from the mono-species cultures could grow in ASM supplemented with 1 mg mL^−1^ colistin (i.e., 250 × MIC); we consider these isolates to be fully resistant to colistin. Perhaps even more strikingly, the prevalence of these fully resistant mutants was highest in the cultures challenged with 2 × MIC_colistin_, indicating that the lower concentration of the drug confers more potent selection for resistance than the higher (5 × MIC) concentration. These “super-resistant” mutants were identified in the samples harvested 1, 8, and 24 h after colistin challenge, but had disappeared by the 48 h sampling point, perhaps indicating that they are associated with a fitness defect in the absence of a selection pressure from the drug. As an aside, we note that we have previously demonstrated that the PA (and SA) in the triple-species steady-state community do not display elevated mutation rates [13]. This suggests that the increased prevalence of mutants resistant to colistin is most likely mediated through selection alone.

We also tested for a difference between the MIC of the *C. albicans* (CA) progenitor strain and the CA isolates collected from the culture vessels challenged with fluconazole (Table 2). Although no extremely high-level resistance (i.e., an MIC > 10 μg mL^−1^) was observed, 26/32 (81%) of the tested CA isolates from the polymicrobial cultures and 16/32 (50%) of the tested isolates from the mono-species cultures were able to grow in the presence of 5 µg mL^−1^ fluconazole (cf. MIC for the progenitor = 1 µg mL^−1^). Once again, we noticed that the highest prevalence of resistance was seen in the cultures treated with the intermediate (2× MIC) concentration of fluconazole. Taken together, our data suggest that growth as part of a polymicrobial community increases the prevalence of mutants that are resistant to the action(s) of some clinically relevant antimicrobial agents.

### Whole-genome sequencing

The high-level resistance to colistin that we observed was intriguing, and in order to pinpoint its genetic basis we used whole-genome sequencing (WGS). Eight *P. aeruginosa* (PA) isolates with MIC > 1 mg mL^−1^ were collected from the continuous-flow cultures challenged with 2× MIC colistin. Two colistin-resistant isolates were collected from the mono-species PA culture after 1 h (isolates A and B) or 8 h (isolates E and F) treatment with colistin, and two resistant isolates were collected from the polymicrobial co-culture after 1 h (isolates C and D) or 8 h (isolates G and H) treatment. As a control, we also re-sequenced an isolate of the progenitor (input) strain of MPAO1 that was used to inoculate each culture.

All sequenced isolates contained an insertion (a single cytosine) adjacent to *dppA1* (PA4496), which encodes a dipeptide ABC transporter substrate-binding protein (Table 3). Although important for the uptake and utilisation of di- and tri-peptides by PA [31], this insertion occurs immediately after the TAG stop codon of *dppA1* and is therefore predicted to have no effect on the functionality or expression of the gene product. All isolates, except D and H, contained SNPs in one of three genes (*pilM*, *pilW* or *pilF*) associated with the biogenesis of type IV pilin. Furthermore, isolates A, D, E, F and H contained SNPs or small indels in *wzy* (PA3154), which encodes B-band O-antigen polymerase. Isolates B and C also contained SNPs in the *wzy* cluster; isolate B contained a SNP in *wbpA* (PA3159) and isolate C contained a SNP in *wbpE* (PA3155). Both of these genes are involved in biosynthesis of the O-antigen oligosaccharide. Isolate C also contained a T → C transition in *ssg* (PA5001), which, although not well-characterised in PA, encodes a putative glycotransferase involved in the biosynthesis of LPS in *Pseudomonas alkylphenolia* [32]. Taken together, our data strongly suggest that colistin resistance in both mono-species and polymicrobial cultures is mediated through mutations in the genes associated with LPS or pilus biogenesis.

**Table 3:** Non-synonymous SNPs and indels associated with the colistin resistant *P. aeruginosa* isolates.

Isolate	Culture type	Time (h)	Locus	Gene	Product	Type	Effect	NT position	AA position
A	Mono	1	PA3154	*wzy*	B-band O-antigen polymerase	snp	missense	1238/1317	413/438
			PA5044	*pilM*	Type 4 fimbrial biogenesis protein	ins	frameshift	531/1065	177/357
			PA4496	*dppA1*	ABC transporter	ins	intragenic		
B	Mono	1	PA3159	*wbpA*	UDP-*N*-acetyl-*D*-glucosamine 6-dehydrogenase	snp	missense	150/1311	50/436
			PA5044	*pilM*	Type 4 fimbrial biogenesis protein	ins	frameshift	531/1065	177/357
			PA4496	*dppA1*	ABC transporter	ins	intragenic		
C	Poly	1	PA3155	*wbpE*	UDP-2-acetamido-2-deoxy-3-oxo-*D*-glucuronate aminotransferase	snp	missense	701/1080	234/359
			PA5044	*pilM*	Type 4 fimbrial biogenesis protein	ins	frameshift	531/1065	177/357
			PA4496	*dppA1*	ABC transporter	ins	intragenic		
			PA5001	*ssg*	Hypothetical protein	snp	missense	569/957	190/318
D	Poly	1	PA3154	*wzy*	B-band O-antigen polymerase	ins	frameshift	129/1317	43/438
			PA4496	*dppA1*	ABC transporter	ins	intragenic		
E	Mono	8	PA3154	*wzy*	B-band O-antigen polymerase	ins	frameshift	129/131	43/438
			PA3805	*pilF*	Type 4 fimbrial biogenesis protein	snp	missense	581/759	194/252
			PA4496	*dppA1*	ABC transporter	ins	intragenic		
F	Mono	8	PA3154	*wzy*	B-band O-antigen polymerase	snp	stop gained	266/1317	89/438
			PA4552	*pilW*	Type 4 fimbrial biogenesis protein	del	frameshift	561/825	187/274
			PA4496	*dppA1*	ABC transporter	ins	intragenic		
G	Poly	8	PA4552	*pilW*	Type 4 fimbrial biogenesis protein	del	frameshift	561/825	187/274
			PA4496	*dppA1*	ABC transporter	ins	intragenic		
H	Poly	8	PA3154	*wzy*	B-band O-antigen polymerase	ins	frameshift	266/1317	89/438
			PA4496	*dppA1*	ABC transporter	ins	intragenic		

Summary of non-synonymous SNPs and indels identified in the genome of PA isolates resistant to the action of 1 mg mL^−1^ colistin when compared with the re-sequenced reference genome of the progenitor (PAO1). All isolates sequenced were collected from a steady-state continuous-flow culture after 1 h or 8 h exposure (as indicated) to 8 µg mL^−1^ colistin (2× MIC). Culture type identifies whether the isolate was obtained from a mono-species (Mono) or polymicrobial (Poly) culture. Mutation type is abbreviated to ins (insertion), del (deletion) or snp (single nucleotide polymorphism). NT position and AA position denote the location of the nucleotide or amino acid change in the gene or encoded protein, respectively.

### Complementation of the colistin-resistant phenotype

**Fig. 5:**
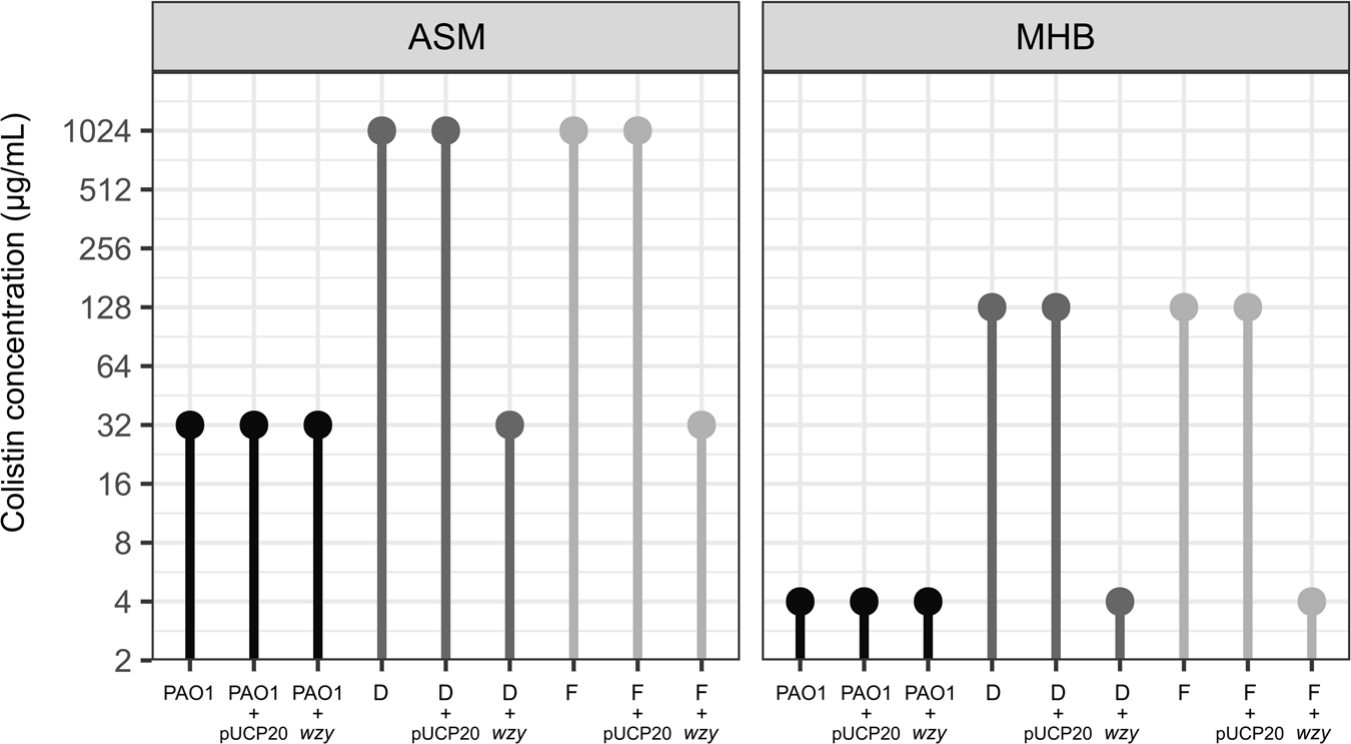
MIC of colistin for colistin-resistant isolates. The minimal inhibitory concentration of colistin is shown for the parental strain (PAO1) and for two of the colistin-resistant isolates (D and F, shown in different shades of grey). The MICs were determined using the microdilution/resazurin method (see methods) for each indicated strain containing either no plasmid, or empty pUCP20, or pUCP20-*wzy*. Experiments were performed using three biological replicates.

In order to test our hypothesis regarding the involvement of the LPS biosynthetic pathway in colistin resistance, we carried out complementation analyses to determine whether susceptibility to colistin could be restored by expression of a wild-type version of the affected gene. Since most (5 out of 8) of the isolates had mutations in *wzy*, we introduced the wild-type version of this gene into the shuttle vector, pUCP20, and expressed this in isolates D and F. These isolates have a frameshift mutation and a premature stop codon (respectively) early on in the *wzy* ORF and therefore likely lead to gross loss-of-function of the gene. As expected, the MIC of isolates D and F for colistin was high in the absence of the wild-type *wzy* gene (Fig. 5). However, the presence of a functional *wzy* (introduced on pUCP20) led to a 32-fold reduction in the MIC_colistin_ in both ASM, and Mueller-Hinton broth (MHB) compared with the isolates transformed with an empty plasmid (Fig. 5). This indicates that the loss of function of *wzy* in these isolates is indeed responsible for the observed colistin resistance phenotype. Taken together, these data demonstrate that the LPS of *P. aeruginosa* plays a crucial role in colistin susceptibility and that loss-of-function mutations in the genes involved in LPS biosynthesis represent a novel mechanism of resistance to this antibiotic.

### Mutations in *wbpA*, *wbpE*, and *wzy* from other *P. aeruginosa* isolates

We next assessed the prevalence of sequence variants in the *wbpA*, *wbpE*, and *wzy* genes from a range of other *P. aeruginosa* strains. To do this, we turned to the International Pseudomonas Consortium Database (IPCD), since this resource comprises *P. aeruginosa* isolates spanning almost 100 years and from a wide variety of isolation sources. Furthermore, all the IPCD genomic sequences have comprehensive associated metadata.

**Fig. 6:**
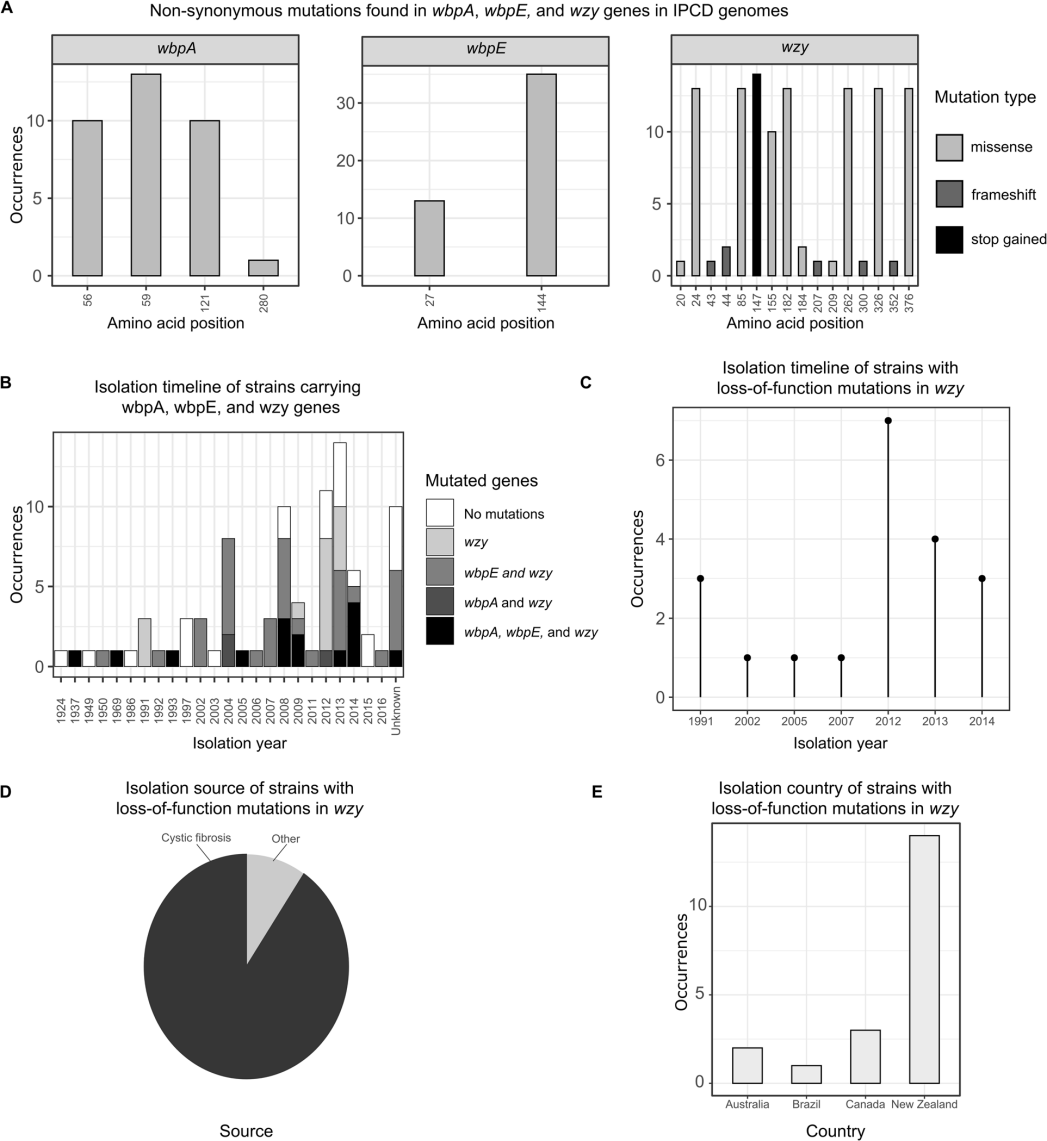
The diversity and prevalence of mutations in O-antigen biosynthesis genes among the IPCD collection. **A** Mutations associated with *wbpA*, *wbpE*, and *wzy*, and their location (amino acid position) in each gene. The different shades of grey represent different types of mutation (missense, frameshift, or nonsense). **B** The number of strains in the IPCD that carry the *wbpA*, *wbpE*, and *wzy* genes, and their year of isolation. The shades of grey differentiate the number of isolates carrying nucleotide variations (relative to PAO1) in the indicated gene(s). **C** Isolation year of the IPCD strains carrying unambiguous loss-of-function mutations in *wzy*. **D** Isolation source of the IPCD strains carrying loss-of-function mutations in *wzy***. E** Geographical origin of the IPCD strains carrying loss-of-function mutations in *wzy*.

First, to ensure that the IPCD isolates are diverse, we carried out Multi-Locus Sequence Typing (MLST) to determine the sequence type (ST) of all the isolates in the database. We found that at least 244 different ST are represented in the 854 strains comprising the IPCD, with 198 strains having an unknown or unidentified sequence type. This confirms that the genomic sequences in the collection are diverse (Supplementary Table S6). We then extracted the *wbpA*, *wbpE*, and *wzy* gene sequences from the IPCD collection and screened these for sequence differences relative to the corresponding PAO1 genes. This revealed 130 single nucleotide sequence variants in *wbpA*, of which 96 led to synonymous changes, and 34 to non-synonymous changes. Similarly, 83 *wbpE* sequence variants were identified; 35 of these led to synonymous changes and 48 to non-synonymous changes. Strikingly, *wzy* was associated with a much higher number (304) of sequence variations than the other genes; 192 of these led to synonymous changes and 92 to non-synonymous changes (Supplementary Table S6). Interestingly, all of the non-synonymous mutations in *wbpA* and *wbpE* were classified as missense variants, whereas in *wzy* we found that in addition to the 92 missense mutations, there were 6 frameshifts and 14 nonsense mutations (Fig. 6A).

Of the 854 genomes comprising the IPCD, 90 encoded the *wbpA*, *wbpE*, and *wzy* genes. Of those 90 isolates, 67 harboured mutations (relative to the PAO1 gene sequences) in *wbpA*, *wbpE*, and/or *wzy*. Most (sixty-five) of these isolates contained at least one non-synonymous mutation in *wbpA*, *wbpE*, and/or *wzy*. The earliest isolate containing the *wbpA*, *wbpE*, and *wzy* genes was harvested in 1924, and contained no mutations relative to the corresponding PAO1 gene sequences. However, from 1937 onwards, most strains harbouring *wbpA*, *wbpE*, and *wzy* exhibited nucleotide sequence variations in one or more of these genes (Fig. 6B). Interestingly, all of the strains that had mutations in *wbpA* or *wbpE* also contained mutations in *wzy* (Fig. 6B). Moreover, of the 90 isolates containing the *wbpA*, *wbpE*, and *wzy* genes, 20 contained unambiguous loss-of-function mutations (i.e., frameshift or nonsense mutations) in *wzy*.

Since our experimental results showed that loss of the genes involved in biosynthesis of O-antigen serotype O5 (the PAO1 serotype) was associated with colistin resistance, we next analysed the metadata associated with the IPCD strains carrying clear loss-of-function mutations (frameshifts and nonsense mutations). These loss-of-function mutations were exclusively associated with *wzy* in the IPCD. The earliest isolate carrying a loss-of-function mutation in *wzy* was from 1991, with most of the remaining loss-of-function *wzy* mutants being collected in the 2010s (Fig. 6C). Most of these loss-of-function mutants were derived from the expectorated sputum of people with cystic fibrosis (Fig. 6D) residing in Oceania (Fig. 6D, E).

## Discussion

In this work, we show a clear decrease in the efficacy of three clinically-used, species-specific antimicrobial compounds (colistin, fusidic acid, and fluconazole) against their target microorganism when that microorganism is grown as part of a steady-state polymicrobial consortium. This observation highlights the need to consider how co-habiting species might alter the response of a principal pathogen to a previously validated therapeutic intervention. Given the diverse polymicrobial communities associated with CF-airway infections, our findings may, in part, explain the failings of current antimicrobial treatment regimens in eradicating *P. aeruginosa* (PA) infections.

Modulations in antimicrobial efficacy in the polymicrobial environment were found to involve physiological (non-heritable) and adaptive (heritable) elements. For example, although growth in the polymicrobial culture appeared to confer protection upon *S. aureus* (SA) against fusidic acid, there was no difference in the antimicrobial susceptibility of the progenitor strain or any recovered isolate following challenge with fusidic acid. This indicates a physiological (non-adaptive) mechanism of tolerance that requires the presence of PA and/or *C. albicans* (CA) in the co-culture. By contrast, a high proportion of the PA or CA isolates challenged with colistin or fluconazole (respectively) displayed heritable resistance against these agents. Interestingly, growth in a polymicrobial environment appeared to increase the frequency of such resistant isolates. WGS analysis of PA isolates resistant to the action of 1 mg mL^−1^ colistin (i.e., 250× MIC) revealed SNPs/indels in seven genes involved in the synthesis of LPS or pilin (*wzy*, *wbpA*, *wbpE*, *ssg*, *pilF*, *pilM*, and *pilW*). Evidence that loss-of-function mutations in *wzy* are specifically responsible for the colistin resistance phenotype was obtained through complementation analyses. Modifications of LPS through addition of 4-amino-L-arabinose to exposed phosphate groups on the lipid A moiety have been previously implicated in conferring resistance to colistin [33]. However, this L-Ara4N modification is associated with a dedicated gene cluster (*arnBCADTEF-pmrE*; [34]. To our knowledge, the current study is the first to prove that mutations in the core LPS biosynthetic pathway genes can also give rise to high levels of colistin resistance. SNPs in genes involved in biosynthesis of type IV fimbrial proteins (pilins) were also found in most of the sequenced PA isolates in this study. Interestingly, mutations in the pilin biogenesis genes have been previously identified in two separate WGS-based studies of colistin-resistant clinical PA isolates [35, 36]. However, and beyond this correlation, there is currently no direct evidence linking pilin synthesis with colistin resistance. Given that isolate G in the current study could grow in the presence of 1 mg mL^−1^ colistin, yet only contains a non-synonymous mutation in *pilW*, this provides strong evidence to suggest that disruption of type IV pilin synthesis can also give rise to colistin-resistance. Interestingly, mutations in LPS and pilin biosynthetic genes are common among clinical PA isolates recovered from late-stage CF airway infections ([37] and Fig. 6).

Colistin was first introduced in the clinic in 1959, but was replaced by aminoglycosides in the 1970s due to concerns over its toxicity [38]. However, colistin saw a resurgence in the late 1980s and early 1990s when it was administered to treat the infections caused by *P. aeruginosa* in the airways of CF patients. Our analysis of the IPCD shows that mutations in the *wbpA*, *wbpE*, and *wzy* genes became increasingly prevalent after 1991, with ~20% of the affected isolates containing clear loss-of-function mutations in *wzy*. [Here, we considered clear loss-of-function mutations to arise from frameshifts and nonsense mutations. Given that missense mutations can also give rise to loss-of-function (as observed for isolates A, B, C, and F in the current study), it seems likely that the actual proportion of loss-of-function mutations is higher than 20%.] Our analysis shows that the first isolates with loss-of-function mutations in *wzy* were collected around the time that colistin was reintroduced into the clinic, in 1991. We appreciate that there is likely to be a significant sampling bias here, especially given that the trend in the number of strains with loss-of-function *wzy* mutations closely reflects the increasing number of isolates collected over time. To reach a more robust conclusion about whether the introduction of colistin to the clinic has had an impact on the selection of mutations in O-antigen biosynthetic genes, a larger genomic dataset will be needed, and this is currently not available.

Our results also provide evidence that species-specific antimicrobial interventions can have unintended consequences on the dynamics of those species that are not targeted by the drug. These consequences are not necessarily predictable either – niche vacation by one species is not necessarily accompanied by niche infiltration by others, although *P. aeruginosa* does appear to be particularly aggressive in this regard. For example, in the polymicrobial populations treated with 5× MIC_fusidic acid_ or 5× MIC_fluconazole_, there was a significant increase in PA cell titres at the 48 h sampling point (compared with pre-treatment titres). This observation supports the climax-and-attack model (CAM) of changes within a polymicrobial community following clinical intervention or immune clearance proposed by Conrad et al[39]. Briefly, this hypothesis suggests that keystone pathogens of late-stage CF airway infections can resist clearance from the airway and acquire adaptations that enable them to occupy a newly liberated environmental niche. This often complicates future treatment regimens and leads to a worsening patient prognosis.

In summary, we show here that growth as a steady-state planktonic polymicrobial community can enhance the resistance of the inhabitants to antimicrobial agents, sometimes, by several orders of magnitude. *P. aeruginosa* was found to be particularly efficient at occupying the niches vacated by other species as a consequence of antibiotic action. Interestingly, exposure to lower (but still lethal, to the progenitor strains) concentrations of antimicrobial agent led to a higher frequency of resistant isolates. This suggests that maintenance of high antibiotic dosages may have hitherto unexpected benefits in terms of suppressing AMR. Finally, we note that the current study does not explicitly examine the impact of growth in a biofilm. Antimicrobial resistance is known to be increased (compared with the planktonic mode of growth) [40] in biofilms. However, we have recently demonstrated that the continuous-flow setup can be used to cultivate polymicrobial biofilms [19] and current efforts are aimed at examining how antibiotic challenge affects the population dynamics in this growth mode too. We are also expanding the species repertoire in the system.

## Supplementary information

Supplemental material

Supplemental material - Bioinformatics analysis

## Data Availability

Sequence reads of the colistin-resistant isolates and progenitor strain were deposited in GenBank under accessions SAMN18614912 to SAMN18614920.
