# Supplementary material for: Decreased efficacy of antimicrobial agents in a polymicrobial environment
Source: ISME J. 2022 Mar 19;16(7):1694–704. doi: 10.1038/s41396-022-01218-7 (PMC9213441; doi:10.1038/s41396-022-01218-7)
Supplement: Supplementary file 1 — Supplemental material [file 41396_2022_1218_MOESM1_ESM.pdf]

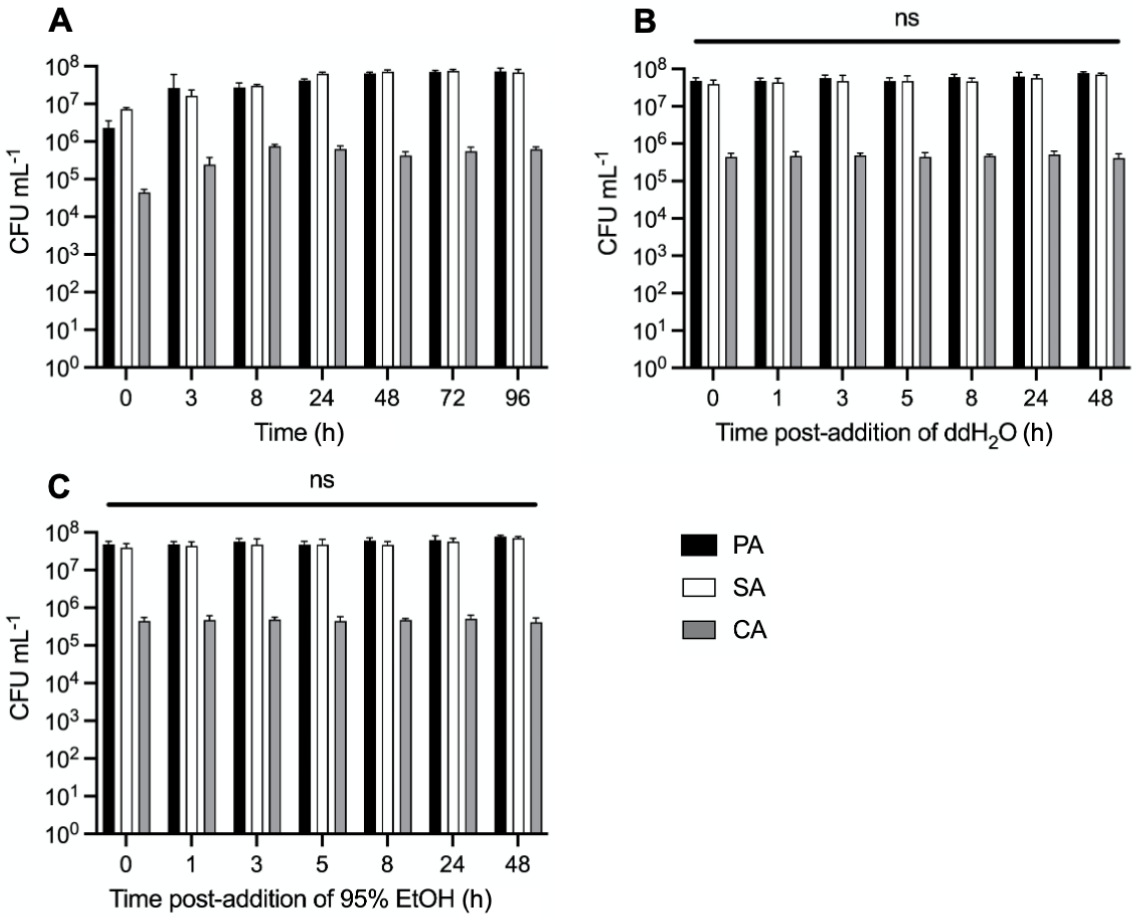

Figure S1. Establishment of steady-state microbial communities and their treatment with antimicrobial solvents. Viable cell counts of *P. aeruginosa* PAO1 (PA, black bars) *S. aureus* 25923 (SA, white bars) and *C. albicans* SC5314 (CA, grey bars) co-cultured in ASM under continuous-flow conditions. Flow rate = 145 µL min^{-1}. **(A)** Polymicrobial cultures reach a steady-state by T = 24 h, following this there is no significant difference in the CFU mL^{-1} counts of any species. Established steady-state polymicrobial cultures (incubated for 24 h) were then treated with **(B)** 1 mL water or **(C)** 1 mL 95% ethanol used to dissolve colistin or fusidic acid/fluconazole, respectively. There was no significant change in CFU mL^{-1} of any species following the addition of the two solvents (at T = 0 h). Data represented as mean ± standard deviation of three independent experiments. CFU mL^{-1} values are plotted on a log_{10} scale and *P* values > 0.05 are considered as not significant (ns).

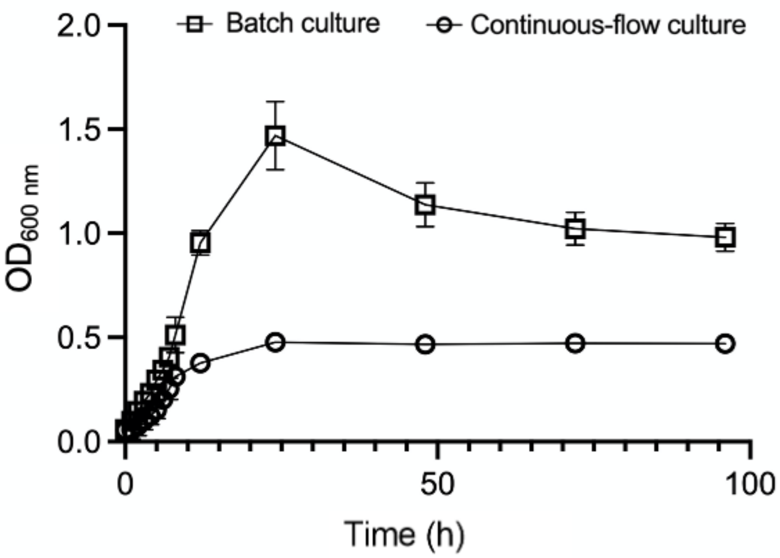

Figure S2. Growth of *P. aeruginosa* PAO1 in ASM in both batch and continuous-flow culture conditions. Growth is monitored by optical density ($OD_{600\ nm}$) during **(A)** continuous-flow culture (Q = 170 µL min^{-1}); **(B)** batch culture (Q = 0 µL min^{-1}). Data represent the mean ± standard deviation from three independent experiments.

Real-time PCR (RT-PCR)

To determine the metabolic state and growth phase of steady-state populations maintained under continuous flow conditions, the relative expression of four stationary phase-specific and four exponential-phase specific *Pseudomonas aeruginosa* genes were selected for RT-PCR from a previously published transcriptomic dataset (1). By targeting eight genes encoding different products differentially expressed between the exponential and stationary phases of growth, discrepancies in the expression of genes due to differences in growth media (artificial sputum media vs AGSY) should be mitigated. Thus, making general trends regarding the metabolic state of the culture obvious. All RT-PCR results were normalised by quantifying the expression of the constitutively expressed 16S rRNA gene encoding for the RNA component of the 30S ribosomal subunit (2-4) and results analysed using the comparative $\Delta\Delta C_t$ method as described by (5). Table S2 shows the target genes and predicted gene products. The reaction mixture and thermocycler conditions used for RT-PCR amplification are provided in Table S3.

RT-PCR primer design

Primer-BLAST software (NCBI, www.ncbi.nlm.nih.gov/tools/primer-blast/) was used to identify suitable primer pairs against the target genes with an approximate T_m of 58°C and yielding an amplicon product of 150 bp.

RT-PCR reactions

RT-PCR amplification was carried out in MicroAmp Optical 96-well Reaction Plates (Applied Biosystems) sealed with MicroAmp Optical Adhesive Film (Applied Biosystems) using a 7300 Real-Time PCR System (Applied Biosystems). Reactions were carried out in 20 µL total volumes, using Universal PowerUp SYBR Green Master Mix (Applied Biosystems), following the manufacturer's instructions and using ROX as a passive reference dye. The reaction mixture and cycle conditions used for all RT-PCR reactions is provided below. RT-PCR of the 16S rRNA housekeeping gene was performed for each cDNA sample tested on every 96-well reaction plate to normalise results (and account for plate-to-plate variability between reactions. Amplicon products were resolved on a 1.4% (w/v) agarose gel to check that a single band of approximately 150 bp in size was present (data not shown).

Primer name	Target gene	Gene product	5' - 3' sequence	Growth phase
rpsM-F	*rpsM*	30S ribosomal subunit protein S13	CGTCGCGAAATCAACATGAAC	*E*
rpsM-R			TTACTTGCGGATCGGCTTAC	
rplM-F	*rplM*	Early assembly protein of the 50S ribosomal subunit	TACCACCACTCCGGCTTC	*E*
rplM-R			CACCTTCAGCTTGCGATACA	
rpoA-F	*rpoA*	DNA-directed RNA polymerase α-chain	GCACCGAAGTGGAACTGTTG	*E*
rpoA-R			CAGTGGCCTTGTCGTCTTTC	
sodB-F	*sodB*	Superoxide dismutase	AACACCTACGTGGTGAACCT	*E*
sodB-R			GCTCAGGCAGTTCCAGTAGA	
rmf-F	*rmf*	Ribosomal modulation factor	ACGGCATAACCGGTAAATCTC	*S*
rmf-R			GCTGGAGTTGATTGAGACGTT	
rsmA-F	*rsmA*	Putative carbon storage regulator	CCCTGATGGTAGGTGACGAC	*S*
rmsA-R			GGTTTGGCTCTTGATCTTTCTCT	
rpoS-F	*rpoS*	Alternative sigma factor	AAGCTCGACCACGAACCTT	*S*
rpoS-R			CGTATCCAGCAGGGTCTTGT	
sodM-F	*sodM*	Superoxide dismutase	CTTCGAGGCGTTCAAGGATG	*S*
sodM-R			ATCGGCGTATTGCCGTTC	
F-16SrRNA-Pa-RT	16S rRNA	RNA component of 30S ribosomal subunit	ACACTGGAACTGAGACACG	*C*
R-16SrRNA-Pa-RT			AGACCTTCTTCACACACG	

Table S1. Target genes and primer pairs designed for RT-PCR analysis of the metabolic state of steady-state microbial cultures.

Forward and reverse primers for a specific target gene are denoted by '-F' or '-R' respectively and growth phase indicates at what point within a typical growth curves these genes are expressed: (E) exponential phase, (S) stationary phase and (C) constitutively expressed.

Reaction mixture		
**Component**	**Volume**	**Final concentration**
2 x PowerUp SYBR Green Master mix	10 µL	
Forward Primer	1 µL	0.25 µM
Reverse Primer	1 µL	0.25 µM
cDNA Template	0.4 µL	10 ng μL^{-1}
Nuclease-free water	7.6 µL	
**Thermocycler conditions**		
**Temperature (°C)**	**Time**	**No. Cycles**
50	2 min	Hold
95	2 min	Hold
95	15 secs	40
60	2 min	
4	∞	Hold

Table S2. RT-PCR reaction mixture and conditions.

Reaction mixture (20 µL total volume) and thermocycling conditions for RT-PCR of target genes using universal PowerUP SYBR Green Master Mix kit and a 7300 Real-Time PCR System.

Primer name	Target gene	Gene product	5’ - 3’ sequence
wzy-F	*wzy*	B-band O-antigen polymerase	ATCCGG *GAGCTC* AGGAGGAACAGCA**ATG**TATAT ACTTGCTCGAGTCGACA
wzy-R			ATCCGG *GCATGC* **TCA**TAGAGTTTTTCCTAAAGAC ATCTTGA

Table S3. Oligonucleotides used for cloning *wzy* in pUCP20. Sequences of forwards and reverse primers to amplify and clone *wzy* in pUCP20. Restrictions sites SacI and SphI are shown in italics, underlined sequences correspond to the ribosome binding site (RBS), and letters in bold represent the start and stop codons.

Reaction mixture		
**Component**	**Volume**	**Final concentration**
5X Q5 Reaction Buffer	10 µL	1x
5X Q5 High GC Enhancer	5 µL	0.5x
Forward Primer	1.25 µL	0.25 µM
Reverse Primer	1.25 µL	0.25 µM
dNTPs	1 µL	0.2 mM
Q5 High-Fidelity Polymerase	0.5 µL	0.02 U/µl
Template DNA	40 ng	0.8 ng μL^{-1}
Nuclease-free water	To 50 µL	
**Thermocycler conditions**		
**Temperature (°C)**	**Time**	**No. cycles**
95	3 min	1
95	15 min	35
57	15 secs	
72	1 min	
4	∞	Hold

Table S4. PCR reaction mixture and conditions for PCR amplification for *wzy*. Reaction mixture (50 µL total volume) and thermocycling conditions for PCR amplification of *wzy* for cloning in pUCP20 using Q5 high-fidelity polymerase.

Species	Minimum inhibitory concentration (μg mL^{-1})		
	Colistin	Fusidic acid	Fluconazole
P. aeruginosa PAO1	4	>256	>256
S. aureus 25923	>256	0.0156	>256
C. albicans SC5314	>256	>256	1

Table S5 Minimum inhibitory concentration of antimicrobial compounds. Minimum inhibitory concentration (MIC, μg mL^{-1}) of colistin, fusidic acid and fluconazole against *P. aeruginosa* PAO1, *S. aureus* 25923 and *C. albicans* SC5314 grown in ASM. MICs were determined using the EUCAST broth microdilution method. The MIC was considered as the lowest concentration of the compound able to inhibit visible microbial growth after 16 h incubation. MIC values were determined using broth microdilution from three independent experiments using different batches of freshly-prepared ASM each time.

Gene	NS/S	Type	Occurrences	Occurrences	Total	Unique strains
wbpA	Synonymous	Synonymous	96	96	130	18
	Non-synonymous	Missense	34	34		
		Frameshift	0			
		Nonsense	0			
wbpE	Synonymous	Synonymous	35	35	83	49
	Non-synonymous	Missense	48	48		
		Frameshift	0			
		Nonsense	0			
wzy	Synonymous	Synonymous	192	192	304	67
	Non-synonymous	Missense	92	112		
		Frameshift	6			
		Nonsense	14			

Table S6 Mutations in *wbpA*, *wbpE* and *wzy* identified in the International Pseudomonas Consortium Database (IPCD). The table shows the number of occurrences of variants, and the type of each variant (synonymous, missense, frameshift or nonsense), in isolates from the IPCD. In all, we identified 67 isolates carrying mutations in *wbpA*, *wbpE* or *wzy*; of these, 65 were associated with non-synonymous variants.

References

1. Mikkelsen H, Duck Z, Lilley KS, Welch M. Interrelationships between colonies, biofilms, and planktonic cells of *Pseudomonas aeruginosa*. J Bacteriol. 2007;189(6):2411-6.
2. Clarridge JE. Impact of 16S rRNA gene sequence analysis for identification of bacteria on clinical microbiology and infectious diseases. Clin Microbiol Rev. 2004;17(4):840-62.
3. Kolbert CP, Persing DH. Ribosomal DNA sequencing as a tool for identification of bacterial pathogens. Curr Opin Microbiol. 1999;2(3):299-305.
4. Woese CR. Bacterial evolution. Microbiol Rev. 1987;51(2):221-71.
5. Giulietti A, Overbergh L, Valckx D, Decallonne B, Bouillon R, Mathieu C. An overview of real-time quantitative PCR: applications to quantify cytokine gene expression. Methods. 2001;25(4):386-401.
